# Training humans for synthetic face image detection

**DOI:** 10.3389/frai.2025.1568267

**Published:** 2025-05-21

**Authors:** Ramlah Sara Rehman, Ewald Meier, Mathias Ibsen, Christian Rathgeb, Robert Nichols, Christoph Busch

**Affiliations:** da/sec—Biometrics and Security Research Group, Hochschule Darmstadt, Darmstadt, Germany

**Keywords:** generative AI, face analysis, synthetic image data, image forensics, biometrics

## Abstract

Fake identities created using highly realistic synthetic face images have become increasingly prevalent in recent years, driven by advancements in generative neural networks that are readily accessible online and easy to use. These fake identities can be exploited for malicious purposes, such as spreading misinformation or committing fraud. Given the widespread availability of online content and the ease of generating fake online identities, it is desirable that users are able to distinguish real face images from synthetic ones. Additionally, it is important to explore whether specialized training can enhance the ability of individuals to detect synthetically generated face images. In this work, we address these challenges by designing an online experiment to evaluate human detection capabilities and the impact of training on detecting synthetic face images. As part of the experiments, we recruited 184 participants divided into an experimental group and a control group, where the experimental group underwent a tailored training session halfway through the experiment. The study shows that training may moderately enhance human capabilities to detect synthetic face images. Specifically, it was found that the experimental group generally outperformed the control group after training, primarily due to improved abilities in detecting synthetic face images. However, after training, the experimental group showed increased sensitivity and misclassified also more authentic face images, as compared to the control group.

## 1 Introduction

Deep learning-based generative models have evolved rapidly in recent years and can produce highly realistic images, raising significant concerns about their potential use for digital deception. Particularly problematic is the generation of realistic synthetic face images, which can be used to create fake identities on social media platforms. These identities can then be used for spam, fraud, and spreading misinformation (O'Sullivan, 2020; Porcile et al., 2024).

The ramifications of synthetic content extend beyond a few isolated instances of fraud, and there is a concern that fake identities and digitally manipulated media (e.g., deepfake videos) can affect public opinion and, for instance, impact public elections (Łabuz and Nehring, 2024; Ulmer and Tong, 2023). Therefore, it is important to develop technical measures capable of mitigating the spread and use of synthetic data for malicious purposes. Consequently, researchers are actively working on detecting fake identities including synthetic and manipulated face images and videos (see e.g., Rathgeb et al., 2022; Tolosana et al., 2020; Nguyen et al., 2022).

Automated algorithms for detecting synthetic face images often focus on identifying distinctive artifacts or differences in face symmetry that may appear in synthetic images (e.g., Matern et al., 2019; Hu et al., 2021) or leveraging learned disparities in the visual or frequency spectra between authentic and synthetic images (e.g., Wang et al., 2020; Ibsen et al., 2024; Zhang X. et al., 2019). However, detecting entirely synthetic face images remains challenging, especially when evaluated under realistic conditions where image impairments may occur (e.g., compression and scaling), or where images generated by a specific model have not been seen during training (Gragnaniello et al., 2022; Rahman et al., 2023). Furthermore, synthetically generated images might be further tampered to deliberately try and fool face manipulation detection algorithms (Neves et al., 2020; Carlini and Farid, 2020). Another issue is that automated algorithms for detecting synthetic face images usually rely on deep learning-based methods and often lack explainability (Tolosana et al., 2022).

Given the widespread exposure to images online, it is crucial to assess how effectively humans can differentiate between synthetic and authentic face images, regardless of the capabilities of automated algorithms. Additionally, it is important to understand whether humans can be trained to enhance their ability to make these distinctions between real and synthetic images. In Nightingale and Farid (2022), the authors showed that it is difficult for humans to distinguish synthetic face images generated by Generative Adversarial Networks (GANs) from authentic images, achieving a close to chance performance of 50%. In the experiment, the participants were tasked with classifying, one at a time, 128 out of 800 faces as being real or synthetic. In the same work, the authors showed that training individuals by exposing them to artifacts that might appear in synthetic face images and giving trial-by-trial feedback slightly improved the overall accuracy, which improved to 59% when considering new participants. Other studies have also shown that humans experience significant challenges in accurately detecting realistic synthetic face images (e.g., Lago et al., 2022; Hulzebosch et al., 2020) and that some face images created by deep learning-based models are perceived as being more authentic than real images; a phenomenon which has been termed AI hyperrealism (Miller et al., 2023).

In the past, the generation of realistic face images was constrained by several factors, including limited access to generative models and training data, the specialized domain expertise required as well as the complex and time-consuming processes involved in configuring and utilizing these models effectively (Tolosana et al., 2020). However, nowadays, realistic synthetic face images can seamlessly be created using various readily available online applications. Therefore, it is important to examine the availability and capabilities of these online applications for generating realistic synthetic face images, and to evaluate human capabilities in detecting images produced by techniques representative of these applications. Additionally, it is important to determine whether specialized training can significantly enhance individuals' ability to detect synthetic face images.

**Figure 1:**
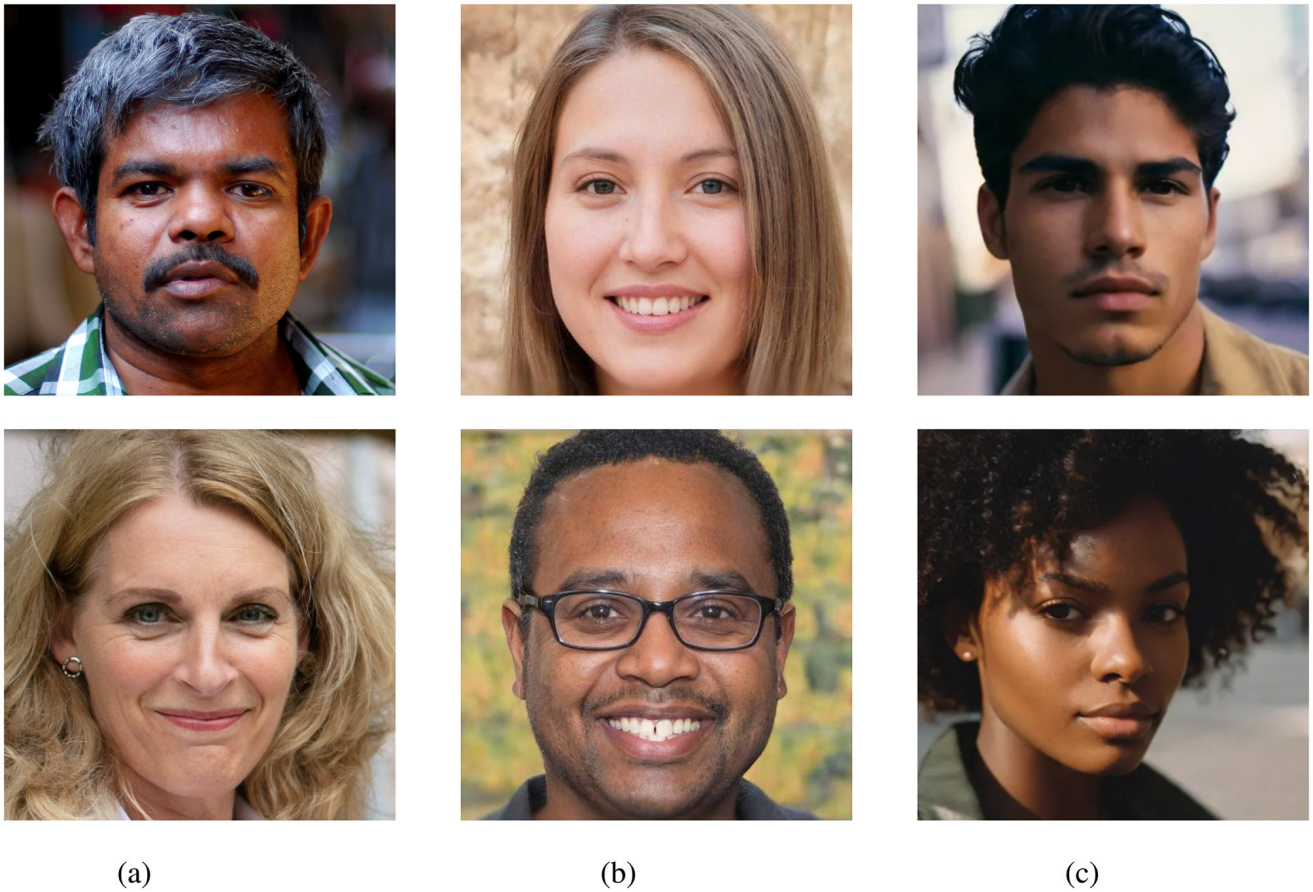
Examples of real and synthetic face images generated using GAN and diffusion-based models. **(a)** Real. **(b)** GAN. **(c)** Diffusion. Figure **(a)** is taken from FFHQ and is CC license: https://github.com/NVlabs/ffhq-dataset?tab=License-1-ov-file#readme.

In this paper, we present an overview of online services for generating synthetic face images and explore the effectiveness of training humans for detecting such fake face images. To this end, we generate a database of realistic synthetic face images employing both GANs and diffusion-based models (see Figure 1). The latter type of technique is gaining popularity in the recent past, but has not been considered in previous investigations on human detection of synthetic face images, e.g., in Nightingale and Farid (2022). Additionally, we design an online experiment to assess whether training can augment human abilities to detect synthetic face images. In this context, it is important to note that previous works on related tasks, e.g., face image manipulation detection (Franco et al., 2023; Godage et al., 2023), did not observe any continual improvement of human examiners' detection capabilities. Contrary to most existing works, we evaluate participant performance through pre- and post-training assessments, comparing results before training and against a control group. This study offers interesting insights into humans' detection capabilities of synthetic face images and their aptitude for learning to distinguish real face images from synthetic ones.

The remainder of this work is structured as follows: Section 2 details the creation of the database utilized in the experiments. The development of guidelines for training individuals to detect synthetic facial images is outlined in Section 3. The experimental design is described in Section 4. Finally, Sections 5, 6 present the results and a summary of the main insights of this work, respectively.

## 2 Dataset creation

The database used for the experiments in this study comprises both synthetic face images and real (pristine) face images. These images were selected to encompass a variety of generation techniques and real images from diverse sources, reflective of portrait images of individuals likely encountered online. Additionally, the face images were post-processed (e.g., aligned) in a consistent manner to minimize discrepancies between the synthetic images from different generation techniques and the real images.

### 2.1 Synthetic face generation

For this study, numerous online services capable of generating synthetic face images were considered. Many of these tools are freely available and easy to use, making them a preferred choice for creating synthetic content. A variety of generation services were considered in this study in order to identify tools appropriate for the generation of synthetic face images. Emphasis was put on tools which allowed for generating diverse and highly realistic face images. It was assessed whether the different generation methods allowed for a customization of the output (e.g., controlling the gender of the subject in a generated image). Furthermore, it was a desired feature that images could be freely generated without restrictions to the number of images that could be created. An overview of generation services which were considered in this work is shown in Table 1. The table presents the degree of customization offered by each tool, their associated costs, and a brief description of each tool. This work does not endorse or promote the use of any of these tools. Instead, the aim was to identify a selection of generation services suitable for this study and representative of various state-of-the-art techniques capable of producing highly realistic face images. For the selection of tools, the criteria in Table 1 were considered (i.e., customization and cost) as well as the realism of the face images generated by these services. While many of the services do not disclose the underlying generation techniques, those that did were primarily based on diffusion or GAN-based techniques. Consequently, two types of tools were selected for this work: a diffusion-based model^1^ and a GAN-based technique built on StyleGAN2 (Karras et al., 2020). Example images generated by the used tools are presented in Figure 1.

**Table 1:** Overview of popular online image generation tools considered for synthetic face generation.

**Tool**	**Customization**	**Cost**	**Details**
aiimagegenerator.org	High	Free	Generates a synthetic image based on a prompt. The generation can be further guided by providing a image or sketch.
openai.com	High	Fee-based	Text-guided generation based on diffusion (see Betker et al., 2023).
deepai.org	High	Fee-based	Offers generation of images based on prompts. Has additional customization options (e.g., generation of HD images).
deepdreamgenerator.com	High	Fee-based	Image generation from a prompt based on different models.
dezgo.com	High	Free^†^	Offers a range of generative models for different tasks including text to image generation models which can be used to generate synthetic face images.
facestudio.app	Medium	Free^†^	Allows generating face images, with some controllable parameters (e.g., pose, gender, and ethnicity).
fotor.com	High	Fee-based	Allows generation of images based on prompts using different models and styles.
generated.photos	Medium	Fee-based	Offers a tool specifically for generation face images with some controllability (e.g., hair color, skin tone, and emotion).
midjourney.com	High	Fee-based	Allows generating images based on textual descriptions. Offers a range of input parameters (e.g., style and aspect ratio).
nightcafe.studio	High	Free^†^	Provides text-to-image generation using models like Flux and Imagen. Supports customizable input parameters such as style and aspect ratio.
stability.ai	High	Free^†^	Offers a range of generative models based on diffusion to generate highly realistic face images.
starryai.com	High	Fee-based	Offers prompt based generation of face images with some extra features (e.g., negative prompts).
thispersondoesnotexist.com	Low	Free	Face image based on StyleGAN2.
this-person-does-not-exist.com	Medium	Free^†^	StyleGAN2-based face generation with control over gender, age, and ethnicity.

Note that some of the fee-based services might offer limited free image generation.

† Images can be generated for free, with optional paid advanced features (e.g., better models or faster generation times).

### 2.2 Real face images

To complete the creation of an appropriate dataset for the evaluation, real face images were collected. These images were collected to match the style of the images generated by the selected GAN and diffusion-based models. Therefore, images from the FFHQ face database (NVIDIA, 2019) were used and enriched by manually selecting high quality professionally-looking face images from free online sources. Example images are shown as part of Figure 1.

### 2.3 Post-processing

To minimize differences between the generative models and the acquisition of real images, both real and synthetically generated face images were aligned using the same technique. Specifically, the alignment method of the FFHQ dataset (see NVIDIA, 2019) was used to align the images, and they were cropped to 512 × 512 pixels. As shown in Figure 1, the eyes in each image are centered in a similar position.

## 3 Human training guidelines

As the study aims to gauge the effect of training on individuals' ability to detect synthetic face images, a training session was created to teach participants specific analysis strategies for helping detect synthetic face images. The training session was delivered only for the members of the experimental group. The training consisted of two parts: (1) a semantic analysis phase where the participants were asked to break down a face and analyze it systematically, and (2) a focus on discovering artifacts and other discrepancies which might occur in some synthetic face images.

### 3.1 Semantic analysis

**Figure 2:**
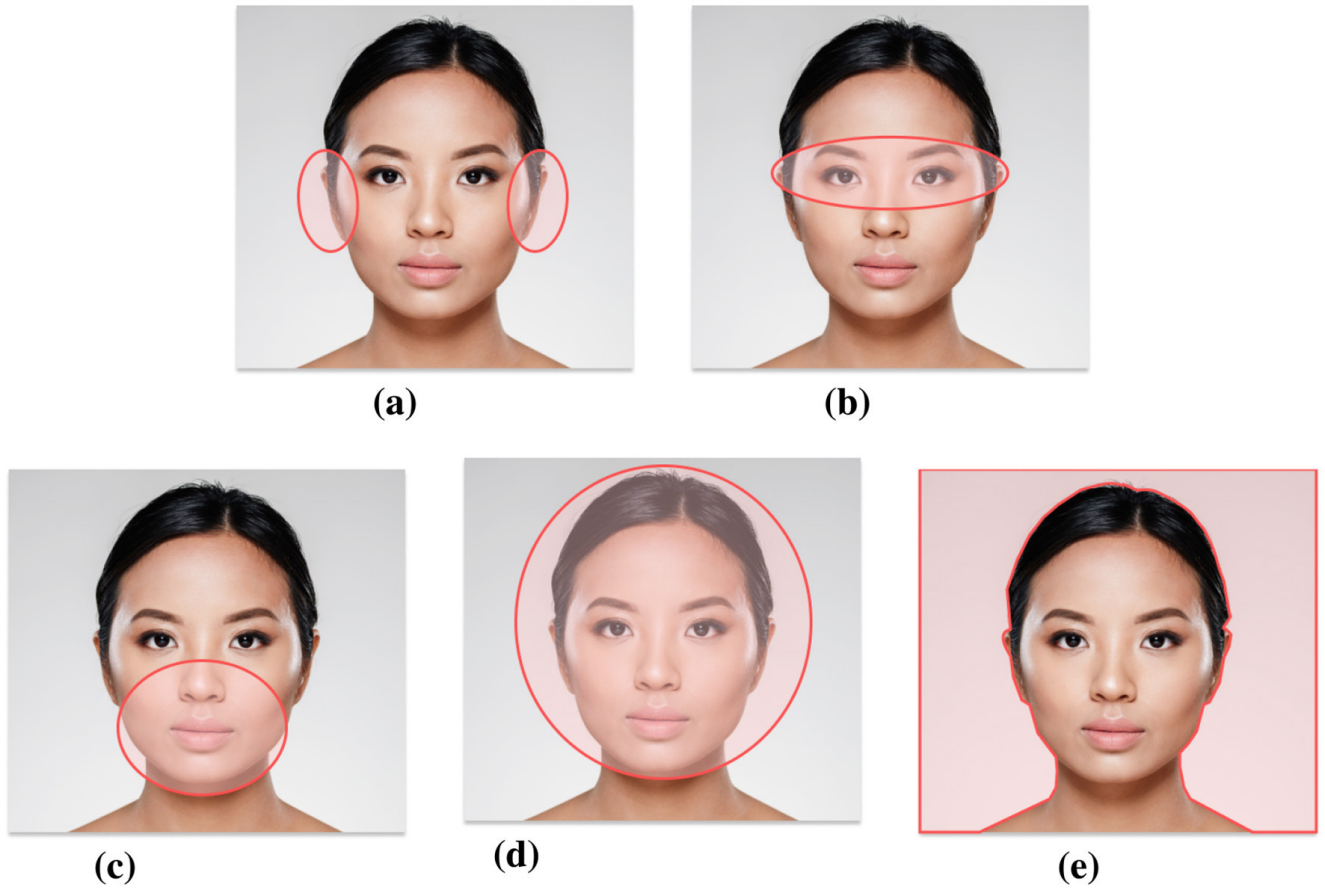
Images provided to participants during the training session, highlighting key facial components to be individually and gradually analyzed. The original image was designed by Freepik (2024), and modified to include highlighted sections. **(a)** Ears, **(b)** eyes, **(c)** lower face, **(d)** forehead and face shape, and **(e)** hair.

In Towler et al. (2021), the authors suggest to recognize faces using a region-based comparison strategy where faces are systematically analyzed based on facial regions which are most diagnostic of identity (e.g., ears and eyes). Particularly, the authors designed a training course where participants were encouraged to break faces into different parts and systematically compare each facial region individually. Additionally, they were taught that some face regions were more useful than others. The authors found that the diagnostic region training could improve accuracy in unfamiliar face recognition by 6%. Inspired by this work, we designed a training session in which participants were taught to systematically break down the face into five parts and analyse them individually: (1) ears, (2) eyes, (3) lower face, (4) forehead and face shape, and (5) hair. The selection of these facial areas is based on Towler et al. (2021) and ranked from most important to least important. Participants received guidelines on how to analyse each region and were provided with illustrations where the relevant facial components were visually highlighted (see Figure 2).

### 3.2 Artifacts and discrepancies

The participants were encouraged to look for artifacts and other discrepancies that may exist in synthetic face images. These potential artifacts and irregularities were collected from a comprehensive review of research material including (Zhang K. et al., 2019; Farid, 2022; Mundra et al., 2023; Rathgeb et al., 2022; Gragnaniello et al., 2022). They were then grouped into categories and described to the participant:

**Texture:** Analyzing the texture of the image and face might help to identify synthetic images. For instance, the participants were asked to note if any texture (e.g., the skin) appeared unnaturally smooth or irregular.
**Facial Symmetry and Proportions:** The participants were asked to analyse if the right and left halves of the face appear asymmetrical or disproportionate, which might be a sign that a image is generated.
**Facial Properties:** The appearance, placement, ratios, or proportions of facial properties may appear inconsistent, distorted, or unrealistic if the image is synthetic.
**Accessories and Makeup:** The appearance of makeup or accessories may be inconsistent or appear unnatural in a synthetic face image. For instance, accessories (e.g., glasses) might appear incomplete as they are not properly generated.
**Visible Objects and Distortions:** A possible indicator of a synthetically generated face image is if unnatural objects or clear distortions appear in the face's background.

**Figure 3:**
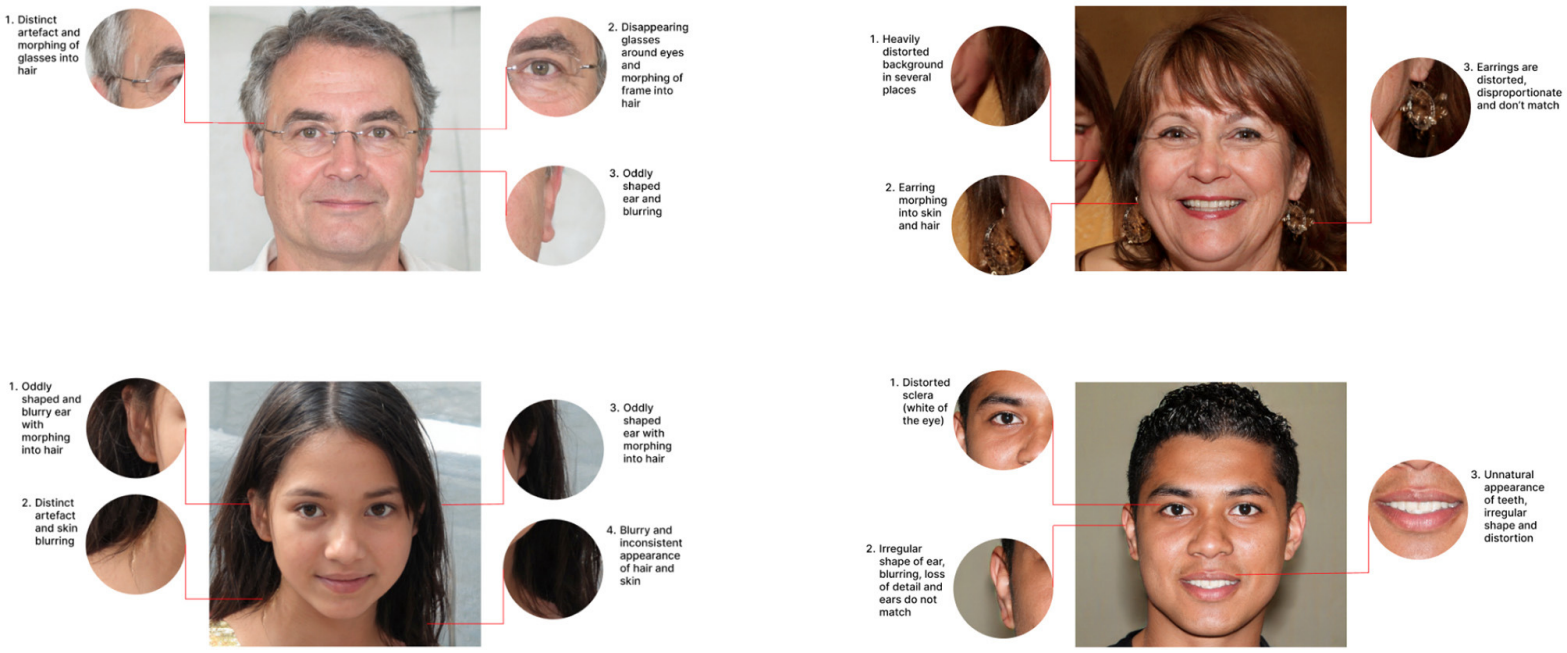
Examples of images with clear visual artifacts or irregularities, where detection clues are visually highlighted and explained to participants during the training session.

The participants were shown example images of various artifacts and irregularities that can occur in synthetic face images. In the first part of the training, the example images focused on highlighting individual artifacts for each of the categories described above. Subsequently, the participants were presented with images containing multiple artifacts or irregularities. Examples of the images shown during this phase of the training are provided in Figure 3. The detailed guidelines are shown in Table 2. We stress that the listed artifacts or discrepancies are potentially observable in synthetic face images generated with current GAN and diffusion-based methods. Hence, it is expected that guidelines need to be adapted for future technologies.

**Table 2:** Categories of artifacts and discrepancies with descriptions.

**Category**	**Description**
Skin texture	Observe that areas of the face or background may appear more or less textured. This means that the skin may appear unnaturally smooth with fine details being lost; it may also include that the entire image has a smooth/blurred effect. The skin may also appear more textured, irregular, or inconsistent.
Facial symmetry and proportions	Note that the right and left halves of the face may appear asymmetrical or disproportionate.
Facial properties	Consider that the appearance, placement, ratios, or proportions of facial properties may appear inconsistent, distorted, or unrealistic.
Accessories and makeup	The appearance of makeup or accessories may be distorted or asymmetrical. For makeup, the appearance of lipstick, eyeliner, and eyelashes may be distorted or misplaced. For accessories, hats, glasses, and earrings appear frequently. Hats may appear disproportionate, blurry, or morphed into the face, skin, or background. The frames of glasses may be partially or completely morphed into the area around the eyes, such that they are indistinguishable. The narrow portion of the glasses frame may be morphed into the ear or the hair. The appearance, shape, size, and symmetry of both earrings may be inconsistent.
Visible objects and distortions	Note the appearance of unnatural objects or artifacts that may appear on the face or background in varying shapes, sizes, and locations.

## 4 Experimental design

**Figure 4:**
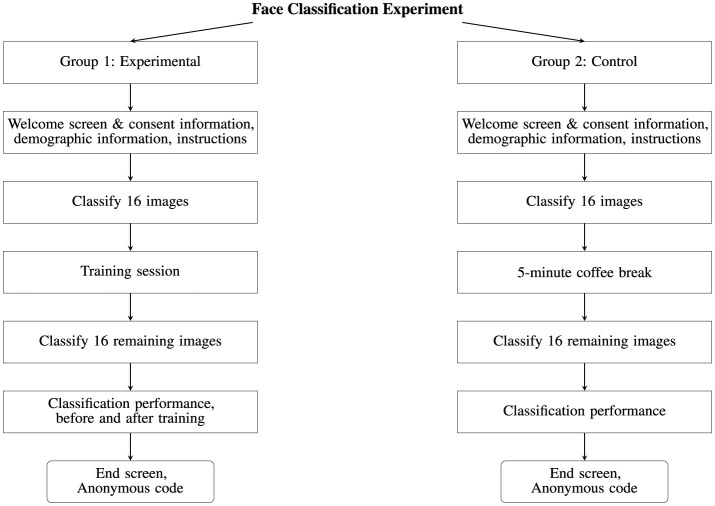
A flow chart depicting a breakdown of the face classification experiment for Group 1 (Experimental) and Group 2 (Control).

The experiments were run online using *PsyToolkit*^2^ which is an online platform designed for conducting psychological experiments and surveys (Stoet, 2016). When accessing the experiments, the participants were randomly split into either the experimental or control group. The participants were then provided with relevant information regarding the experiment, their rights as participants and the information that would be collected. All participants provided explicit confirmation of their participation in the online experiments, affirming their understanding and agreement with the experiment's participant information sheet and consent form. Each participant was asked to self-report their age, gender, and ethnicity (see Section 4.3). The data used in this work has been collected in accordance with the provisions of the General Data Protection Regulation (GDPR) (European Parliament and Council of the European Union, 2016) and is based on explicit consent. No personal identifying information (e.g., name or e-mail address) was stored as part of the experiments; instead, each participant was provided with an anonymized code associated with their collected data. An overview of the experimental design is shown in Figure 4.

### 4.1 Experimental groups

To evaluate the impact of training on the participants' ability to detect synthetic face images, they were split into two groups:

**The Experimental Group:** The participants received training halfway through the experiment.
**The Control Group:** The participants received a 5-minute coffee break halfway through the experiment but no training.

For both experiments, all stimuli and experimental factors were kept the same, with the only difference being that one group received training and the other received a 5-min optional coffee break. Structuring the experiments in this way allowed for two points of comparison: The first point of comparison included comparing participant accuracy scores before and after they had received the training. The second point of comparison was made between the experimental and control group. In this way, the effects of training could be measured both within subjects and between subjects (Charness et al., 2012).

### 4.2 Procedure

**Figure 5:**
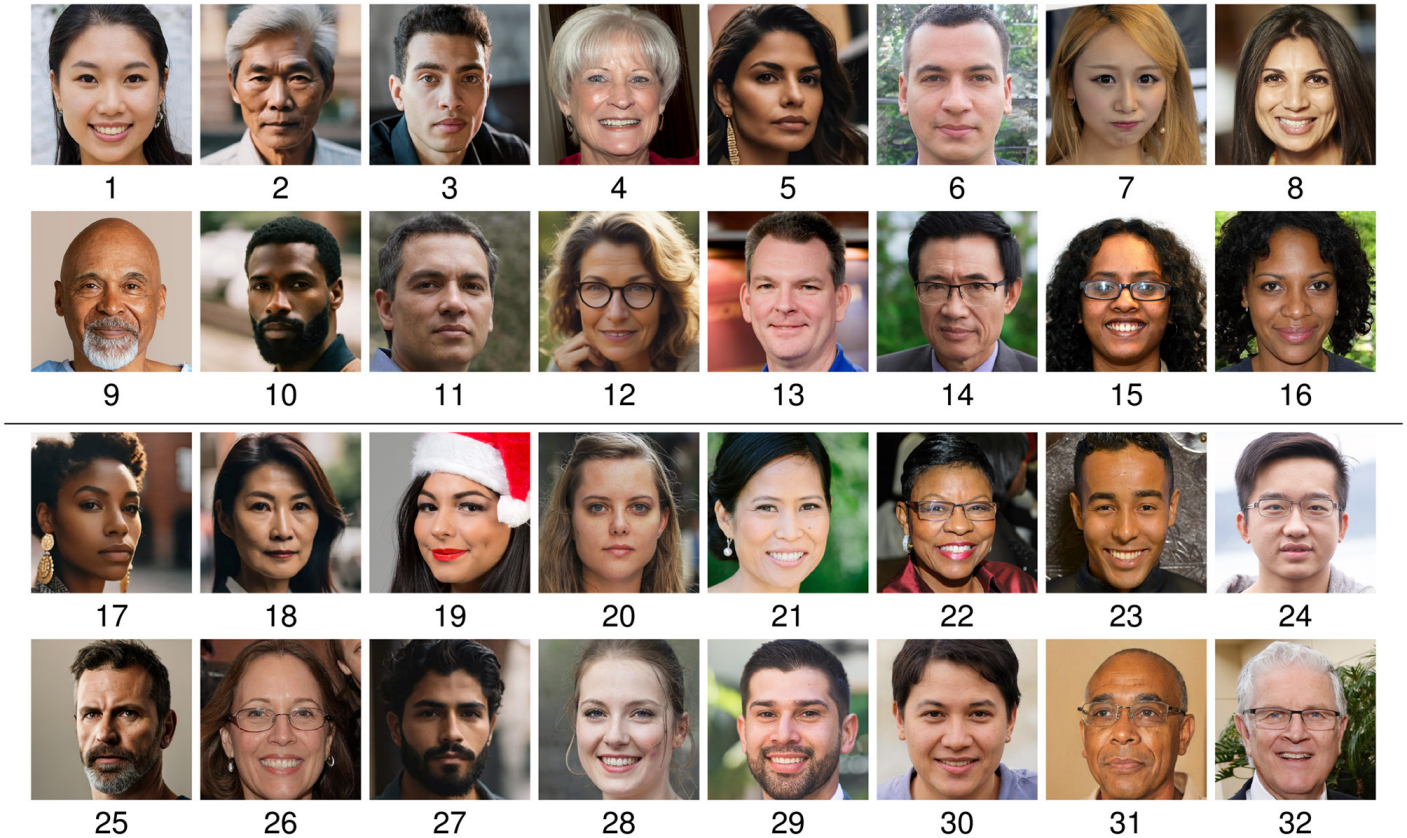
Trial images shown to the participants from top left to bottom right before and after the training/break.

**Figure 6:**
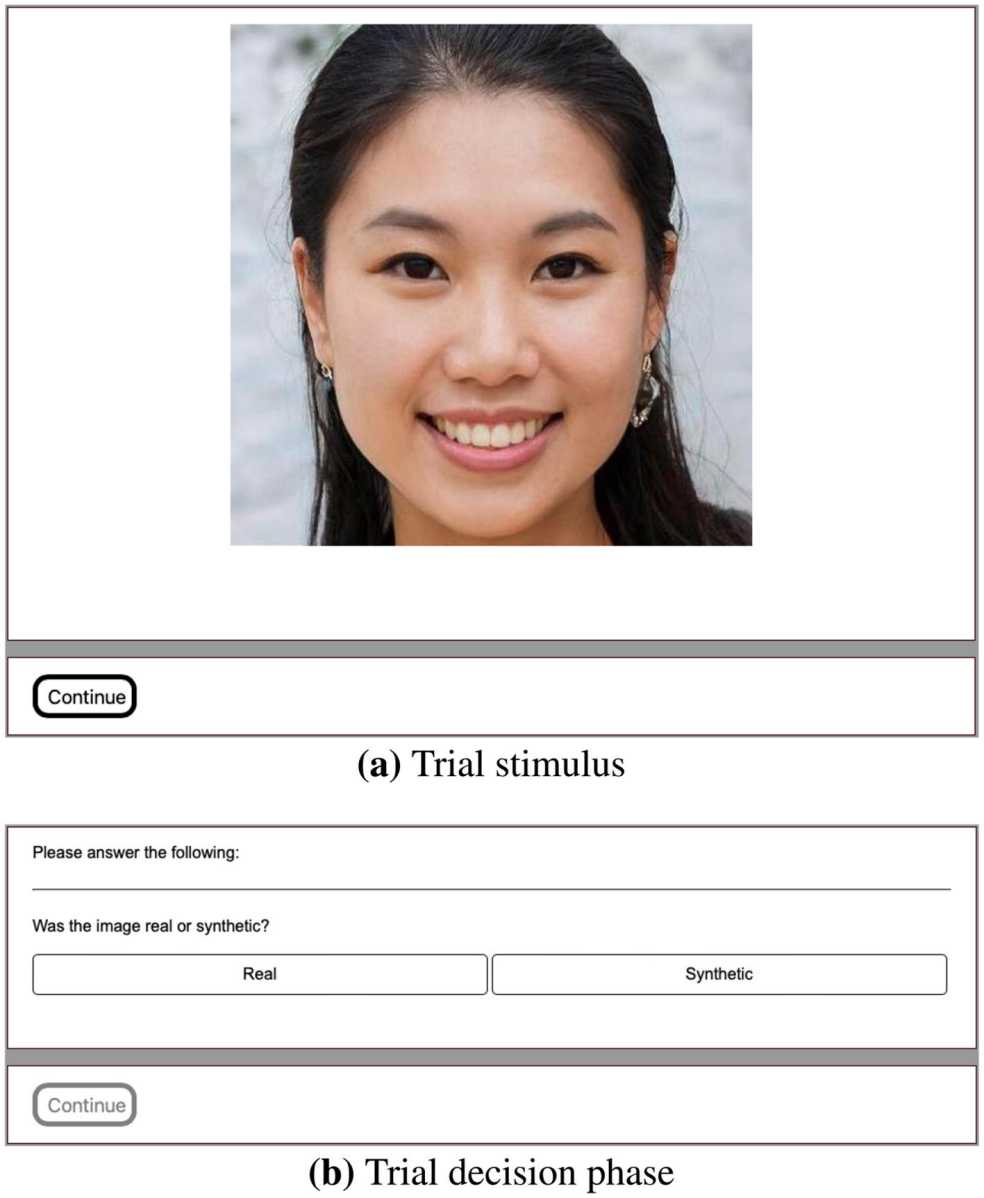
Example from the online test. **(a)** Trial stimulus containing a potentially synthetic face image and **(b)** the trial voting phase.

The experimental procedure consisted of a total of 32 trials. For each trial, the participants were shown a single stimuli (i.e., a real or synthetic face image) for maximum 15 seconds whereafter the participants were asked to select whether the image was real or synthetic, see Figure 5. An example of a trial is shown in Figure 6. The trials were balanced such that in half the trials, the visual stimuli consisted of a real face image, whereas in the remaining trials, they were synthetically generated face images. The order of the trials was determined semi-randomly, ensuring an equal number of real and synthetic face images for each half of the experiment, meaning eight real and eight synthetic images both before and after the training session or coffee break. Additionally, the images were selected to ensure that the real and synthetic face images included in the experiment were balanced across ethnicity and gender. This was achieved by automatically labeling the ethnicity (i.e., Asian, Caucasian, Hispanic, or Black) and gender of the identities depicted in the real and synthetic face images.

Once participants had classified 8 and 24 images, they were prompted to take an optional 30 second screen break where they were encouraged to look away from the computer screen and at a distant object. The purpose of the breaks was to provide a comfortable experience for participants and to possibly reduce the risk of eye strain (Menaria et al., 2024).

After having classified 16 images, participants in the experimental group were directed to a training session based on the guidelines outlined in Section 3. Conversely, participants of the control group were instead given the option to take a 5 min coffee break. Upon completing the 32 trials, the participants were presented with their classification performance, their anonymized code, and a final debrief screen.

### 4.3 Participants

To determine the number of participants required for the study, including the number of participants in the control and experimental groups, a G*Power analysis^3^ (Faul et al., 2007, 2009) was conducted. The sample size calculation was conducted using a t-test to compare the means of two independent groups, with the aim of detecting a difference between experimental group participants and control group participants in their ability to identify synthetic faces. The parameters included a one-tailed test, an effect size (d) of 0.5, an alpha error probability (α) of 0.05, and a power (1 - β error probability) of 0.95, with an allocation ratio of 1:1. The calculation determined that each group should consist of 88 participants, resulting in a total sample size of 176. The calculation is based on the expectation that training participants will improve their detection capabilities, as suggested by previous research (Hulzebosch et al., 2020), and assumes a medium effect size with equal sample sizes in both groups. The input parameters used for the analysis are summarized in Table 3.

**Table 3:** Input parameters for G*Power analysis to calculate the ideal sample size for the experiment.

G*Power analysis parameters	
**Test family**	t tests
**Statistical test**	Means: difference between two independent means (two groups)
**Input parameter**	**Value**
Tail(s)	One
Effect size (*d*)	0.5
α error probability	0.05
Power (1-β error probability)	0.95
Allocation ratio (*N*_2_/*N*_1_)	1

After the ideal sample size was determined for both the experimental and control groups, participants were recruited from various sources, including social media platforms, the European Association for Biometrics (EAB) through their newsletter, a university campus in Denmark, as well as student and professional groups in both Denmark and Germany. The data was collected between April and June of 2024. Table 4 provides an overview of the survey participation and the number of participants assigned to the experimental and control groups. As the experiment was conducted online, it was accessible to a wide segment of the population. Table 5 shows the demographic distribution of the participants. It is observable that certain demographic (sub-)groups are underrepresented, for instance elderly people and black or African American.

**Table 4:** Overview of survey completion and group assignment.

**Participation overview**	**Count**
Survey completion	184
**Group assignment**	
Experimental group	95
Control group	89

**Table 5:** The demographic distribution for the participants.

**Category**	**Groups**	
	**Experimental**	**Control**
**Age distribution**		
18–24	43.2%	36.0%
25–34	34.7%	39.3%
35–44	7.4%	12.4%
45–54	5.3%	5.6%
55–64	5.3%	5.6%
65+	2.1%	0.0%
Prefer not to answer	2.1%	1.1%
**Sex distribution**		
Female	43.2%	44.9%
Male	53.7%	53.9%
Non-binary	2.1%	0.0%
Prefer not to answer	1.1%	1.1%
**Ethnicity distribution**		
American Indian or Alaska Native	1.1%	0.0%
Asian	20.0%	25.8%
Black or African American	0.0%	3.4%
Hispanic or Latino	6.3%	6.7%
White	62.1%	56.2%
Other	7.4%	4.5%
Prefer not to answer	3.2%	3.4%

A total of 254 initiated the online experiment, the final dataset comprises data from 184 of these participants. Data from participants were excluded if they did not complete the entire experiment or if they requested deletion of their data after the experiment.

## 5 Results

**Figure 7:**
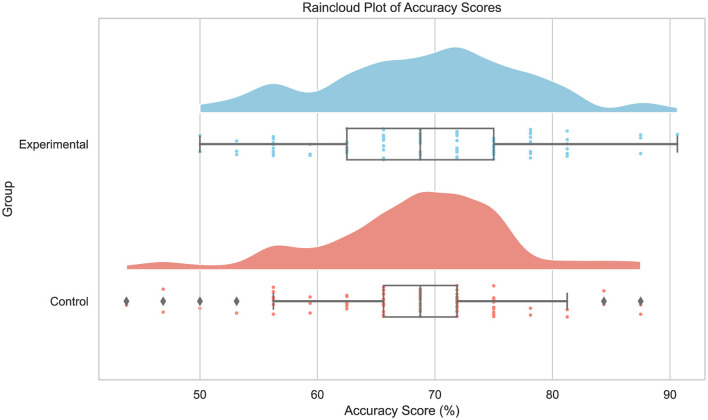
Raincloud plots depicting the distribution of accuracy scores for the experimental and control group.

D'Agostino-Pearson test for normality was conducted to verify that detection accuracy is normally distributed. Results indicate that this is the case for both experimental (*p* = 0.88) and control (*p* = 0.06) groups. Additionally, Levene test results show the assumption of equal variances holds (*p* = 0.31) as well. The detection accuracy for the experimental and control group is shown in Table 6 and Figure 7. The average detection accuracy of the experimental group is 69.28%, whereas it is 68.22% for the control group, indicating a 1.06 percentage point improvement in the experimental group, *t*_(182)_ = 0.83, *p* = 0.41. However, observing in more detail the accuracy of the two groups before and after training in Table 6, it can be noted that the experimental group improves by approximately 3.69 percentage points (i.e., 67.43 to 71.12), *t*_(94)_ = −2.57, *p* = 0.01. In contrast, the improvement for the control group was only approximately 0.21 percentage points (i.e., 68.12 to 68.33), *t*_(88)_ = 0.16, *p* = 0.88. After training, the experimental group performed on average approximately 2.79 percentage points (i.e., 71.12 vs. 68.33) better than the control group, *t*_(178.9)_ = 1.76, *p* = 0.08. Since the conditions of the two groups were kept the same except the training session, these results indicate that the training session did improve, yet not significantly, the overall classification performance of the participants in the experimental group. Notwithstanding, their improvement when comparing the accuracy on the first 16 images with the remaining images was far greater than the control group which received no training. This, and the fact that the prevalence of real and synthetic face images, as well as those generated by each generative model, was the same before and after the training session or coffee break, warrants a more detailed examination of performances.

**Table 6:** Summary statistics of accuracy scores (%) obtained in the experiment.

**Group**	**Mean**	**Median**	**Std. Dev**.	**Min**	**Max**
**Experimental group**					
Before training	67.43	68.75	10.59	43.75	100
After training	71.12	75.00	11.82	43.75	93.75
Overall	69.28	68.75	8.78	50.00	90.62
**Control group**					
Before break	68.12	68.75	11.27	43.75	100
After break	68.33	68.75	9.69	43.75	87.50
Overall	68.22	68.75	8.40	43.75	87.50

**Figure 8:**
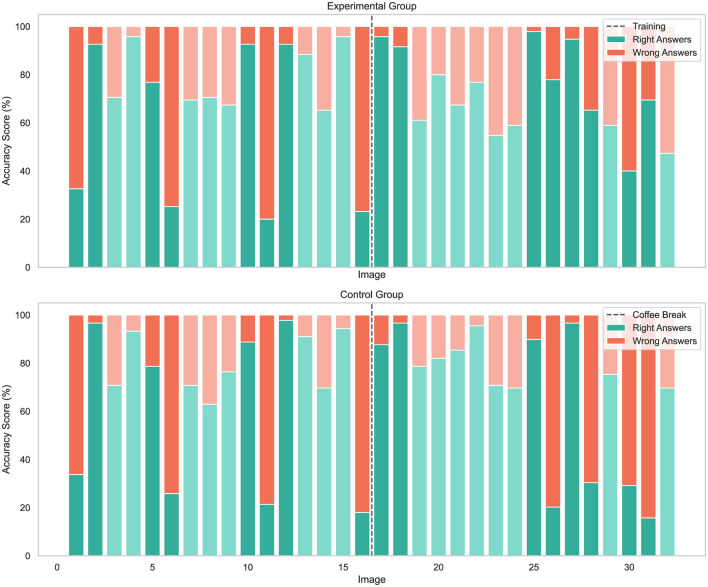
Accuracy results per image for the experimental and control group. The vertical gray dotted line indicates the midpoint where the experimental group gets training and the control group has a coffee break. The darker shades of green and red represent the cases where the stimuli is a synthetic face image, while the lighter shades indicate real images. Trials 1, 6, 11, 16, 26, 28, and 30–31 contain GAN-generated synthetic images, while trials 2, 5, 10, 12, 17–18, 25, and 27 include diffusion-based synthetic images.

Figure 8 shows a more detailed break down of the correct and wrong classifications for each trial stimulus in chronological order. Interestingly, the accuracy for both the control and experimental groups follows a similar trend across the first 16 images for both real (mean 78.65, SD 17.29; and mean 77.89, SD 15.73) and synthetic face images (mean 57.58, SD 14.91; and mean 56.97, SD 16.07). However, after the training session, the experimental group demonstrated significantly higher, *t*_(182)_ = 8.41, *p* < 0.001, classification accuracy for synthetic images (mean 79.08, SD 18.36) than the control group (mean 58.29, SD 14.83). At the same time, their performance on real images (mean 63.16, SD 19.92) declined when compared to the control group, *t*_(182)_ = −5.37, *p* < 0.001, for which performance remained nearly unchanged (mean 78.37, SD 18.44). This suggests that the training enhances the ability to detect synthetic images, albeit with an adverse effect on identifying real images. A closer look at the performance achieved on the synthetic images prior to any intervention reveals that for experimental and control groups alike, GAN-generated images appear harder to detect (mean 25.26, SD 28.36; and mean 24.72, SD 27.82) than diffusion-based images (mean 88.68, SD 20.55; and mean 90.45, SD 16.21). Interestingly, considering post-intervention performances, the experimental group improved on synthetic GAN image detection (mean 63.16, SD 32.38) compared to the control group (mean 23.88, SD 29.65), *z* = 269.0, *p* < 0.001. Nevertheless, this improvement does not appear to extend to the performance on diffusion-based images for experimental and control groups (mean 95.00, SD 14.40; and mean 92.70, SD 15.63), *z* = 112.5, *p* = 0.09.

These findings suggest a training advantage for challenging images specific to the type of generation approach, however the small number of images in this type of comparison represents a limitation, thus low statistical power prohibits definitive claims. Although results indicate a greater challenge in detecting GAN-generated images over diffusion-based images, this can only be stated for the present test configuration. At most, a momentary indication can be derived for the singular diffusion system used and does not allow for statements regarding diffusion approaches in general, even more so considering the rapid development of this diverse technology.

**Figure 9:**
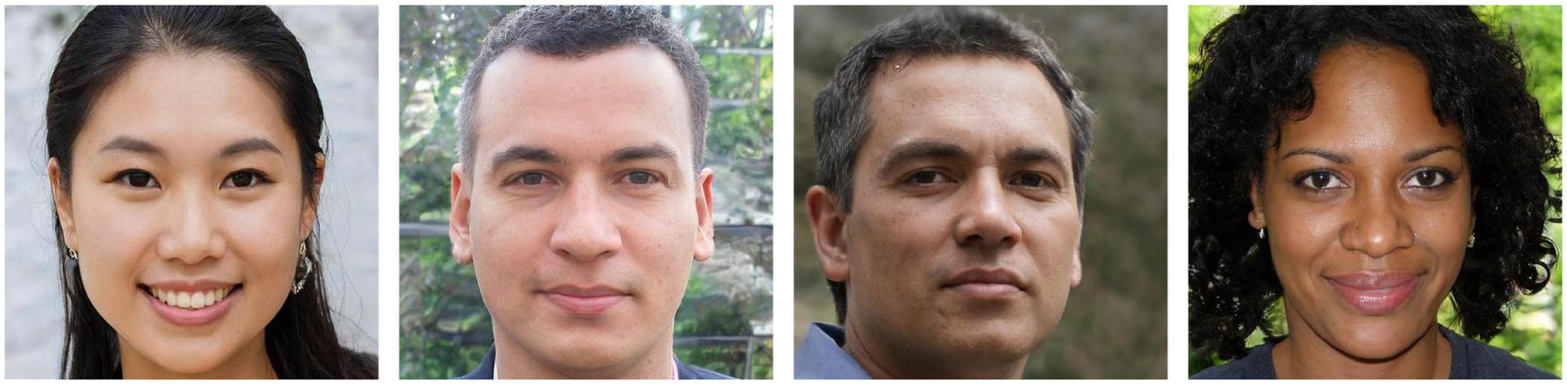
Examples of synthetic face images (GAN) which were difficult to detect by both the experimental and control groups.

Figure 9 shows examples of synthetic images which were difficult for both the experimental and control groups to detect. Precisely, this means that the participants generally achieved low accuracy score on the images. The images shown in Figure 9 correspond (from left to right) to image 1, 6, 11, and 16 in Figure 8, which are all GAN-generated.

## 6 Conclusion and future work

This paper introduced a new online experiment for investigating if humans can benefit from training when detecting entirely synthetic face images in an online setting. To this end an online experiment was designed and an appropriate dataset of real and synthetic face images were collected. A total of 184 participants were recruited to take part in the experiments and were randomly assigned to be part of either the experimental or the control group. The results showed that before a training phase, both the experimental and control group achieved similar detection accuracies whereas after a short training session the experimental group performed, on average, 2.79 percentage points better than the control group who did not receive this training. Furthermore, the average detection accuracy of the experimental group improved by 3.69 percentage points when compared to its accuracy prior to training. Moreover, it was found that the training appeared to improve the ability to detect synthetic face images but had a negative impact on identifying real ones.

Future work could explore the training effect on specific generative models in more detail and evaluate the effect when considering more participants and experimental procedures with varying pre-valences of real and synthetic face images. Such work would ideally be prepended by an extensive evaluation on image fidelity, covering the wide range of generation models to eliminate biases and determine a balanced and challenging set of state-of-the-art synthetic face images. Since the proposed training improved the detection performance, they might as well be used to improve the synthetic face generation technologies. As mentioned earlier, the demographic distribution of the participants of the conducted experiments is rather unbalanced. That is, recruiting participants with the goal of obtaining demographically balanced groups could be subject to future work. Moreover, while this work considered a “One-Shot” training protocol, repeated training sessions could be investigated in the future. Also, it would be interesting to show participants the same 16 images before and after training, and test if they alter their classification. Finally, it would be interesting to compare the detection accuracy of humans to those of state-of-the-art synthetic image detection models.

## Data Availability

The raw data supporting the conclusions of this article will be made available by the authors, without undue reservation.

## Footnotes

^1^ https://dezgo.com

^2^ https://www.psytoolkit.org/

^3^ https://www.psychologie.hhu.de/arbeitsgruppen/allgemeine-psychologie-und-arbeitspsychologie/gpower
